# The efficacy and safety of vonoprazan–amoxicillin dual therapy in eradicating *Helicobacter pylori*: a systematic review and meta-analysis

**DOI:** 10.1186/s40001-023-01249-6

**Published:** 2023-08-07

**Authors:** Jia-Hui Feng, Jie Cheng, Yao-Jia Lao, Kai Huang, Juan-Li Mou, Fan Hu, Meng-Lu Lin, Jun Lin

**Affiliations:** 1 Department of Gastroenterology/Hepatology, Zhongnan Hospital of Wuhan University, Wuhan, China; 2 The Hubei Clinical Center & Key Laboratory of Intestinal & Colorectal Diseases, Wuhan, China

**Keywords:** *Helicobacterpylori*, Vonoprazan, Amoxicillin, Dual therapy, Meta-analysis

## Abstract

**Aim:**

To evaluate the efficacy and safety of vonoprazan–amoxicillin (VA) dual therapy for radically eradicating *Helicobacter pylori* (*H. pylori*).

**Methods:**

The PubMed, Cochrane Library, Embase, China National Knowledge  Infrastructure (CNKI) and Wanfang databases were searched up to July 7, 2022, to identify clinical trials comparing the efficacy of VA dual therapy and triple therapy for *H. pylori* eradication. After evaluating the quality of the included studies, random effects models were conducted, and risk ratios (RRs) with 95% confidence intervals (CIs) were calculated to estimate the efficacy and safety of each approach.

**Results:**

Six publications (including four randomized controlled trials) involving 2019 patients were included in this meta-analysis. Overall, the eradication rate for VA dual therapy was 89.9%, while it was 85.2% for triple therapy based on other acid inhibitors. The eradication rate of *H. pylori* in the VA dual regimen group was higher than that in the PPI-based (omeprazole or lansoprazole) triple therapy group (RR = 1.15, 95% CI 1.07–1.23, p < 0.0001). However, the efficacy of VA dual therapy was comparable with VA–Clarithromycin (VAC) triple therapy (RR = 0.97, 95% CI 0.93–1.02). Besides, the incidence of adverse reactions in VA dual therapy was also lower than that in triple therapy (RR = 0.80, 95% CI 0.70–0.91, p = 0.0009).

**Conclusion:**

Compared with PPI-based triple therapy, VA dual therapy showed a better therapeutic effect, safety and patient compliance rate for eradicating *H. pylori*, which should be used as a novel curative strategy in the future.

**Supplementary Information:**

The online version contains supplementary material available at 10.1186/s40001-023-01249-6.

## Introduction

There are approximately 4.4 billion individuals with *Helicobacter pylori (H. pylori)* infection worldwide in 2015 [1]. *H. pylori* is closely related to gastric cancer, peptic ulcers, and some nervous system diseases [2, 3]. Once *H. pylori* is eradicated, the recurrence rate will be extremely low, which is beneficial for reducing the prevalence of *H. pylori* and related diseases [4, 5]. The current international clinical guidelines recommend quadruple therapy involving proton pump inhibitors (PPIs), two antibiotics, and bismuth as the first-line treatment for *H. pylori*. However, the large number of medicines will not only lead to poor patient compliance but also result in gut microbiota dysbiosis [6]. Recently, antimicrobial resistance has received an increasing amount of attention. Therefore, it is necessary to develop a new solution that requires fewer antibiotics but is more effective in eradicating *H. pylori*.

Vonoprazan is an orally bioavailable potassium-competitive acid blocker (P-CAB) developed by Takeda that has been approved in Japan for the treatment of acid-related disorders [7]. Although vonoprazan is only indicated for reflux oesophagitis in China, many clinical trials around the world have examined the use of vonoprazan-based triple therapy to eradicate *H. pylori*. A large number of studies have reported that vonoprazan-based triple therapy is better than traditional PPI-based triple therapy [8–10]. In October 2018, Sho Suzuki initiated the clinical trial of vonoprazan combined with amoxicillin (VA) dual therapy (vonoprazan 20 mg + amoxicillin 750 mg bid). However, there was no significant difference in eradication rates between VA and PPI-based triple therapy (87.5% vs. 90.2%, *p* = 0.372), and the adverse reaction rate was quite similar [14]. Another study showed that patients in dual therapy had positive results after treatment (93.5% vs. 83.9% *p* = 0.042) [16]. With the advantage of less dosage and potential curative effect, VA dual therapy is expected to become a novel method for eradicating *H. pylori,* but its efficacy and safety are still controversial. Therefore, the current meta-analysis aims to provide robust findings on the efficacy and safety of VA therapy for *H. pylori* by examining the current state research.

## Materials and methods

### Data sources and searches

This meta-analysis was performed in accordance with the PRISMA statement [11]. The PubMed, Embase, Cochrane Library, China National Knowledge Infrastructure (CNKI), and Wanfang databases were searched up to July 7, 2022. Additionally, the reference lists of the included studies were manually searched. Both MeSH and the free-text terms “vonoprazan”, “VPZ”, “potassium competitive acid blocker”, “*helicobacter pylori*” and other associated words were included. The search strategies were provided in the Additional file 1.

### Literature inclusion and exclusion criteria

Only studies that were published in English or Chinese were considered. The additional inclusion criteria were as follows: (1) clinical trial of VA dual therapy for *H. pylori* eradication; (2) the control group was triple therapy based on acid inhibitors; (3) the research subjects were confirmed to have *H. pylori* infection by urea breath test, *H. pylori* antibody or endoscopy and pathology; and (4) the outcome of the study included the *H. pylori* eradication rate and incidence of adverse reactions (nausea/vomiting, bloating, etc.). The exclusion criteria were as follows: review studies, conference studies, abstract-only studies, and low-quality studies assessed by evaluation tools.

### Data extraction

Two researchers independently screened the titles, abstracts and full texts of the obtained literatures, excluding studies that did not meet the inclusion criteria, and cross-checked by another researcher. Reaching a consensus through discussion or soliciting the opinions of a third researcher was required if we had disagreement. The data, including first author, publication year, sample size, research design, and outcome indicators, were all extracted.

### Quality assessment

The Cochrane Risk of Bias Assessment Tool was used to assess the quality of randomized controlled trials (RCTs) across the following domains: selection bias, performance bias, detection bias, attrition bias, reporting bias, and other bias [12]. The Newcastle‒Ottawa scale (NOS) was utilized to assess the quality of the other studies [13]. The total score of the NOS ranged from 0 to 9, with 7–9 points indicating high research quality. Publication bias was assessed by examining funnel plots.

### Statistical methods and data analysis

Meta-analysis was performed using R4.1.1 software, and the heterogeneity test among the studies was carried out by the Cochrane’s Q test. The fixed effects model was used for meta-analysis when there was no statistical heterogeneity among the studies (*p* > 0.05 and *I*^2^ ≤ 50%). If there was statistical heterogeneity among the studies (*p* ≤ 0.05 or *I*^2^ > 50%), the random effects model was applied to account for the potential bias caused by heterogeneity. The risk ratio (RR) and the corresponding 95% confidence interval (CI) were calculated to determine the efficacy of VA dual therapy. The Chi-square test was used to analyze the differences in eradication efficacy of VA groups based on different duration and dosage.

## Results

### Study selection and basic characteristics

**Fig. 1:**
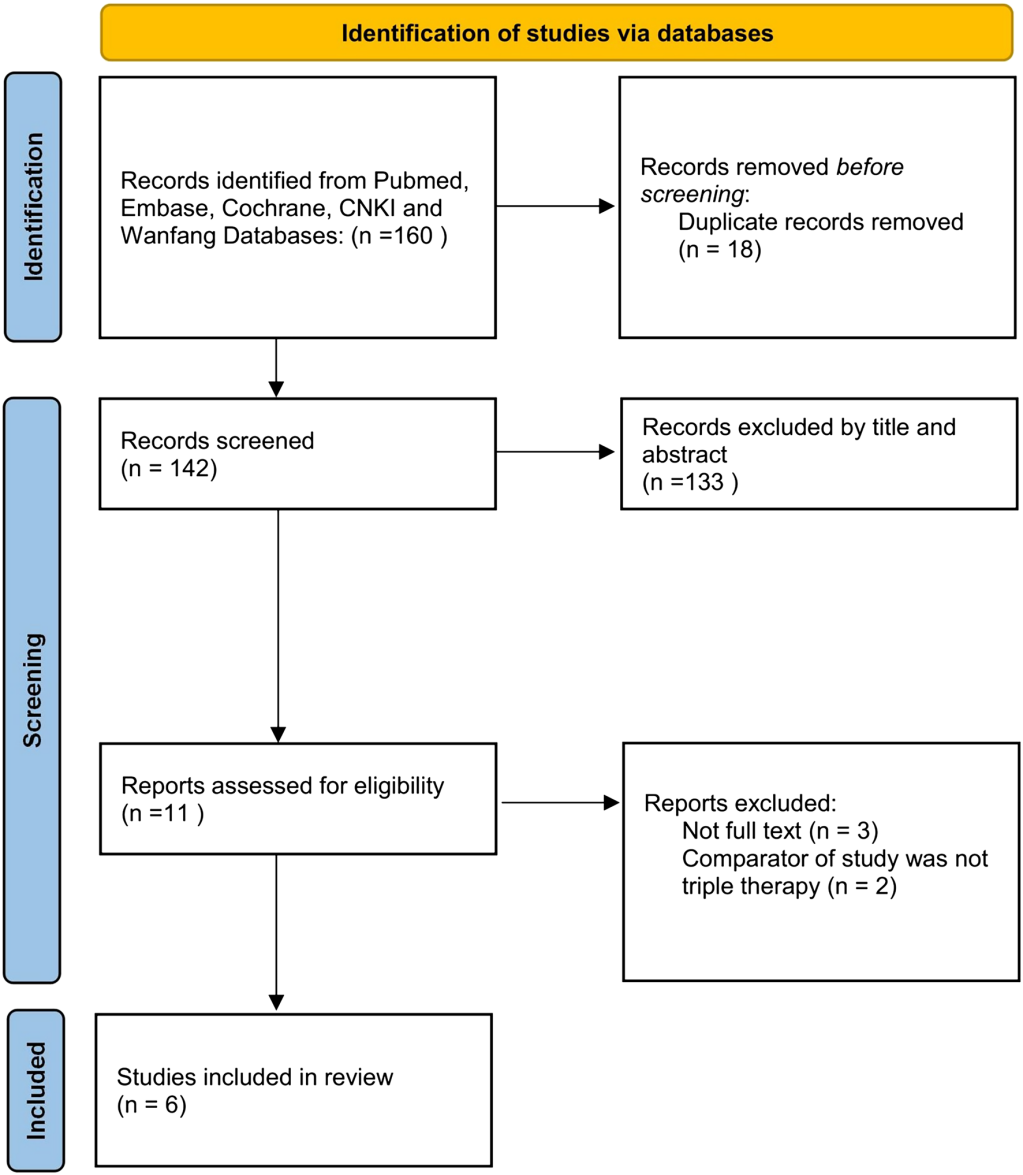
Flowchart of the details of the study

As shown in Fig. 1, a total of 160 studies were retrieved, including 145 studies from the English databases and 15 from the Chinese databases. Six studies were ultimately included for analysis based on the inclusion and exclusion criteria.

The six included studies involved a total of 2019 patients [14–19]. Four studies were RCTs, and two studies were not. The experimental groups were treated with the VA dual regimen, and the control groups were treated with triple therapy based on acid inhibitors. The outcome of *H. pylori* eradication was confirmed by the urea breath test. The basic characteristics of the studies included in our analysis were shown in Table 1.

**Table 1:** Basic characteristics of included studies

Study	Type of study	Patients number	Experimental group	Control group	Eradication rate(E,Con)	Adverse effect(E,Con)
Zuberi et al. [16]	RCT	179	V20mg, bid; A1g bid; 14 days	O20mg, bid; A1g, bid; C500mg, bid 14 days	93.5% (86/92) 83.9% (73/87)	13.0% (12/92) 37.9% (33/87)
Jian Chen [19]	RCT	100	V20mg, qd; A1g bid; 14 days	L30mg, bid; A1g, bid; C500mg, bid; 14 days	98.1% (52/53) 85.1% (40/47)	0.06% (3/53) 0.04% (2/47)
Chey [15]	RCT	542	V20mg, bid; A1g tid; 14 days	L30mg, bid; A1g, bid; C500mg, bid; 14 days	81.1% (215/265) 70.0% (194/277)	29.9% (104/348) 34.5% (119/345)
Suzuki et al. [14]	RCT	327	V20mg, bid; A750mg, bid 7 days	V20mg, bid; A750mg, bid; C200mg, bid 7 days	87.1% (142/163) 90.2% (148/164)	27.3% (46/168) 30.5% (51/167)
Gotodaet al. [18]	Prospective pilot study	216	V20mg, bid; A750mg, bid 7 days	V20mg, bid; A750mg, bid; C200mg, bid 7 days	86.4% (51/59) 84.1% (132/157)	10.0% (6/60) 19.9% (32/161)
Furuta et al. [17]	Retrospective study	109	V20mg, bid; A750mg, bid 7 days	V20mg, bid; A750mg, bid; C200mg, bid 7 days	94.4% (51/54) 92.7% (51/55)	16.1% (9/56) 25.0% (14/56)

RCT: randomized controlled trial; V: vonoprazan; A: amoxicillin; O: omeprazole; L: lansoprazole; C: clarithromycin; E: experimental group; Con: control group

### Quality assessment of inclusion

**Fig. 2:**
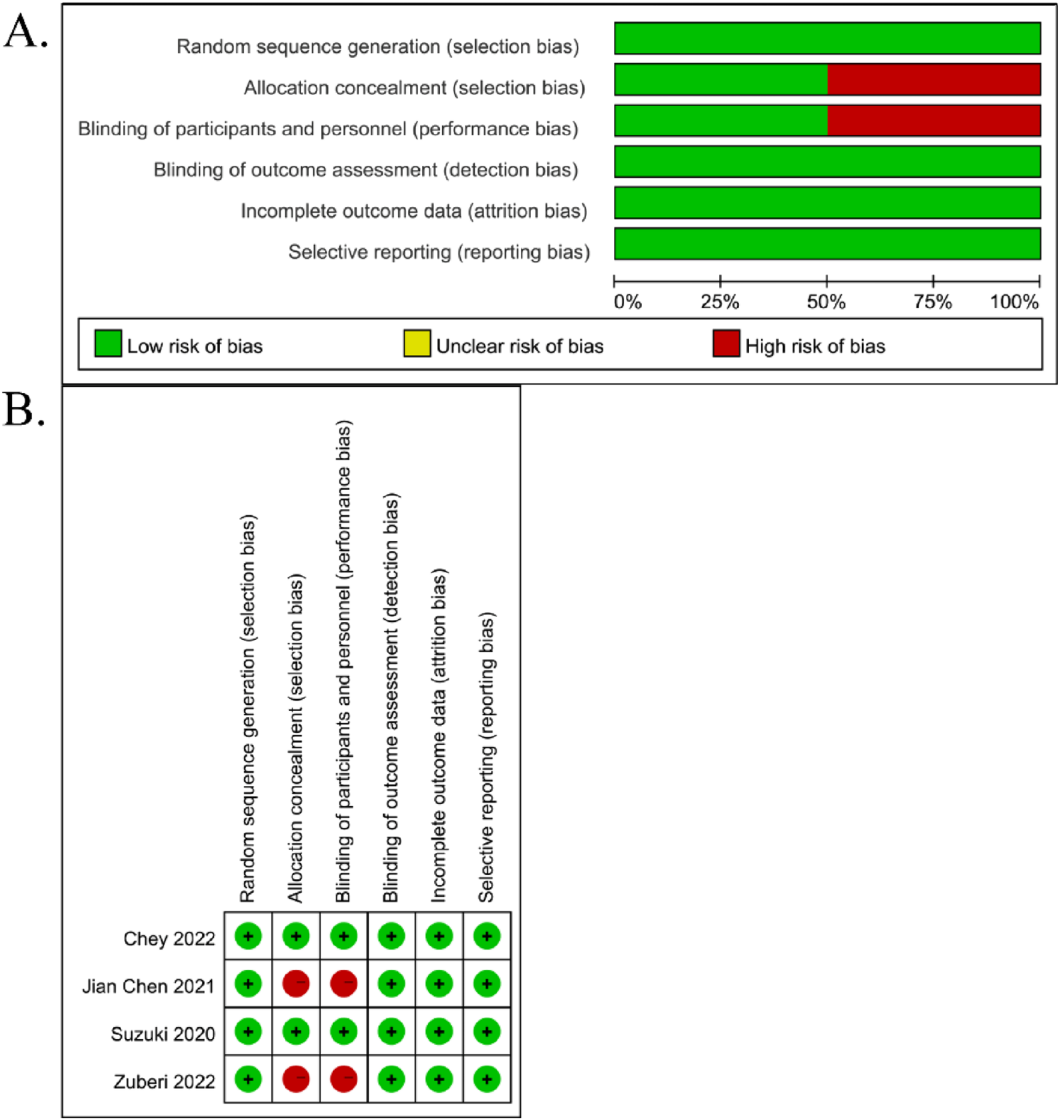
Quality assessment of RCT studies

There were four RCTs and the results of risk bias were summarized in Fig. 2. Zuberi et al. [16] and Chen et al. [19] did not adopt the methods of blinding and allocation concealment, which were difficult to achieve under the nature of interventions. The risk bias of non-RCTs was assessed by NOS and shown in Table 2. The total scores of NOS were 6 and 7 in the research of Furuta et al. [17] and Gotoda et al. [18], respectively. Overall, most of the studies had a low risk of bias.

**Table 2:** NOS scores of two non-RCTs studies

**Study**	Q1	Q2	Q3	Q4	Q5	Q6	Q7	Q8	Total NOS score
Furuta et al. [17]	1	0	1	0	1	1	1	1	6
Gotoda et al. [18]	1	0	1	1	1	1	1	1	7

Q1: representativeness of the exposed cohort; Q2: selection of the non-exposed cohort; Q3: ascertainment of exposure; Q4: outcome of interest not present at the start of the study; Q5: comparability of cohorts; Q6: assessment of outcome; Q7: follow-up long enough; Q8: adequacy of follow-up of cohorts. The NOS assigns up to a maximum of nine points for the least risk of bias in three domains

### Efficacy evaluation of VA dual therapy

**Fig. 3:**
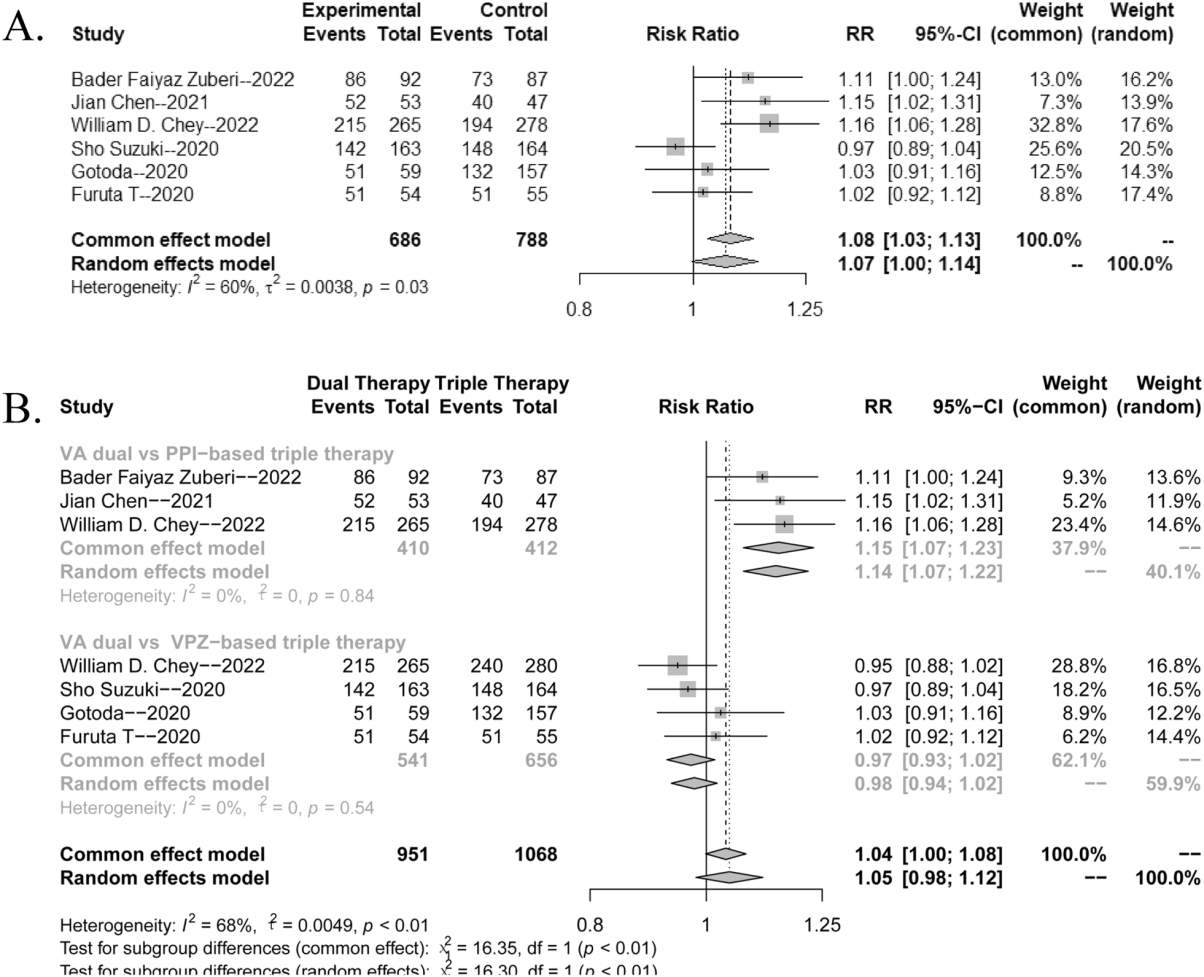
Forest plots for the *H. pylori* eradication rate of VA dual therapy

As shown in Fig. 3A, there was heterogeneity among the studies (*p* = 0.03, *I*^2^ = 59%); therefore, the random effects model was adopted for the meta-analysis. The forest plot results implied that the eradication rate of *H. pylori* in the VA dual regimen group was higher than that in the triple therapy group (RR = 1.07, 95% CI 1.00–1.14).

Based on the selection of control group (triple therapy of acid inhibitors) in the included studies, we divided this meta-analysis into two groups to explore more reliable results. As shown in Fig. 3B, there was no significant heterogeneity among the studies (*p* = 0.84 or 0.54, *I*^2^ = 0%). Therefore, the common effects model was adopted for the following meta-analysis. We found that the eradication rate of *H. pylori* in the VA dual regimen group was higher than that in the PPI-based (omeprazole or lansoprazole) triple therapy group (RR = 1.15, 95% CI 1.07–1.23, *p* < 0.0001). Conversely, the forest plot results implied that VA dual therapy was comparable with VA–Clarithromycin (VAC) triple therapy (RR = 0.97, 95% CI 0.93–1.02).

As shown in Table 3, there were no significant differences between 7-day (244/276, 88.4%) dual therapy and 14-day (353/410, 86.1%) dual therapy (*χ*^2^ = 0.778, *p* = 0.378). The low-dose (20 mg qd) vonoprazan group (52/53, 98.1%) was superior to the high-dose (20 mg bid) (301/357, 84.3%) group (*χ*^2^ = 54.48, *p* < 0.0001).

**Table 3:** Comparison of different doses and treatment courses of VA therapy

	Success	Failure	χ^2^	p-value
Treatment courses				
7 days	244	32		
14 days	353	57	0.778	0.378
Doses of VPZ				
20 mg qd	52	53		
20 mg bid	301	56	54.48	< 0.0001

### Safety evaluation of VA dual therapy

**Fig. 4:**
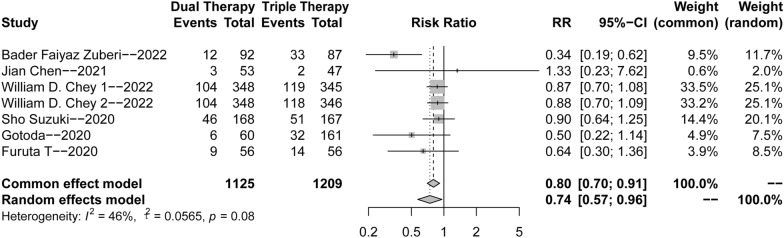
Forest plots for the adverse reactions of VA dual therapy. The control group were PPI-based triple therapy (Chey 1) and VAC therapy (Chey 2) separately

Due to the low degree of heterogeneity (*p* = 0.08, *I*^2^ = 46%), the common effects model was used for the meta-analysis of safety. As expected, the results indicated that the incidence of adverse reactions in the VA dual therapy group was lower than that in the triple therapy group (RR = 0.80, 95% CI 0.70–0.91) (Fig. 4).

### Sensitivity and subgroup analysis

**Fig. 5:**
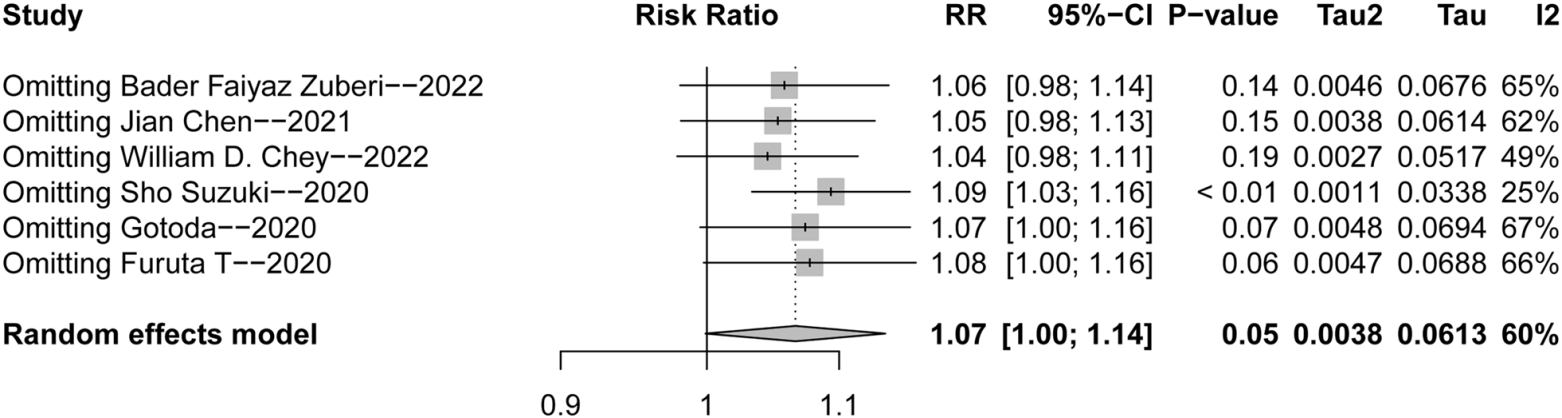
Forest plots for sensitivity analysis

**Fig. 6:**
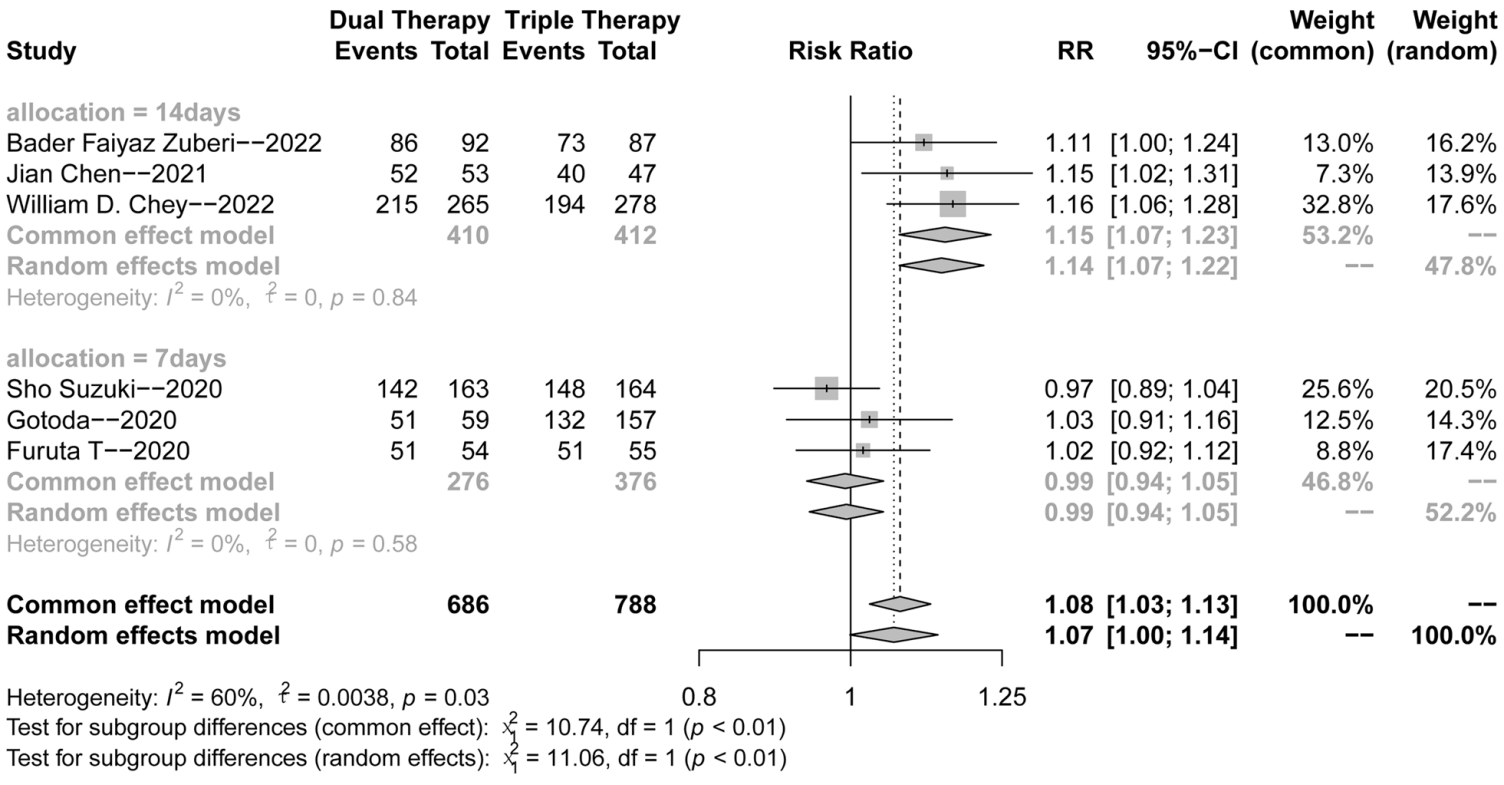
Forest plots for subgroup analysis (14 days vs 7 days)

**Fig. 7:**
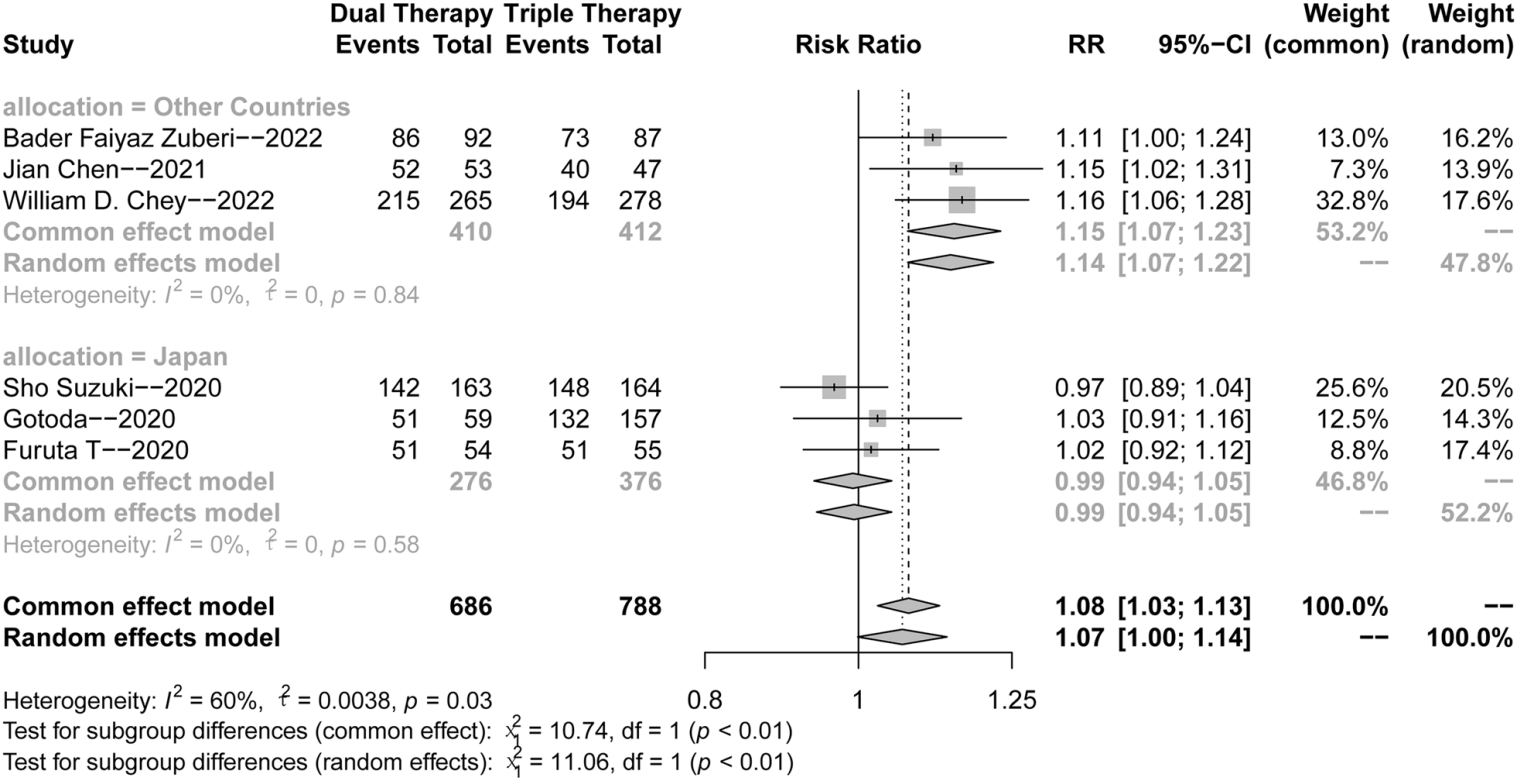
Forest plots for subgroup analysis (from Japan vs. from other countries)

The sensitivity analysis (Fig. 5) revealed that after eliminating the first three studies, the combined effect size crossed the invalid line. We found that the treatment duration of the first three clinical trials was 14 days, while that of the last three clinical trials was 7 days, which might be a possible source of heterogeneity. Hence, we classified the treatment time into 14-day and 7-day groups. Subgroup analysis (Figs. 6 and 7) was performed, and the heterogeneity was significantly decreased, thereby supporting the hypothesis that the course of treatment and study site might be the sources of heterogeneity.

### Publication bias

**Fig. 8:**
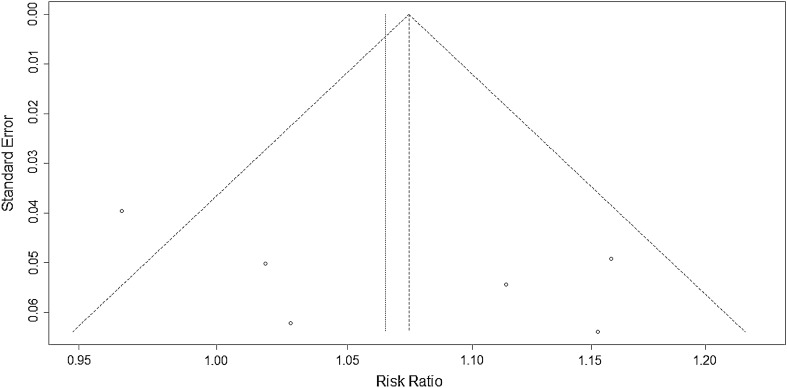
Funnel plot of publication bias

Publication bias was analyzed with RR as the abscissa. The standard error of each study was drawn as a funnel plot (Fig. 8) on the vertical axis. Even though the results revealed that the funnel plot was roughly symmetrical, the degree of publication bias was still uncertain due to the small number of included studies.

## Discussion

Due to the increasing antimicrobial resistance of *H. pylori*, the clinical effectiveness of PPI-based regimens has been reduced, and the failure rate of these approaches ranged from 60 to 70% [20, 21]. Alternative approaches that have been investigated include substituting vonoprazan for PPIs, adding probiotics, and developing vaccines. In addition, based on the genomic, proteomic and metabolomic analysis of *H. pylori*, narrow-spectrum antibiotics and new therapeutic targets can be identified to achieve the effect of individualized treatment [22].

Since vonoprazan entered the market in 2015 as an anti-*H. pylori* drug, Hideki Mori et al. retrospectively analyzed the data of 4097 patients using vonoprazan and found that the *H. pylori* eradication rate had significant improvement (78.5–90.1%) [23]. VA dual therapy has shown acceptable efficacy and safety in the treatment of *H. pylori* infection, and some research has shown that the effective rate of VA dual therapy may be related to the surface area of the human body [28]. VA dual therapy can avoid unnecessary antibiotic use, thereby potentially avoiding antimicrobial resistance [24]. Additionally, the effect of VA dual therapy on intestinal flora is weaker than that of other treatments [25, 26]. No serious adverse reactions occurred in adults and middle school students [18], indicating that VA dual therapy is a safe approach to eradicate *H. pylori*. In addition, traditional PPIs need to be taken on an empty stomach to increase their effectiveness. Studies have reported that the relative bioavailability of vonoprazan is not affected by an empty stomach or eating, which will yield more choices and better compliance for patients [27].

In this study, four RCTs and two non-RCTs were included, and the efficacy and safety of VA dual therapy for eradicating *H. pylori* were systematically evaluated. VA dual therapy has been considered as a potential treatment due to its curative effect and low adverse reactions. It is natural that more antibiotics can achieve a higher eradication rate. However, in this meta-analysis, the efficacy of VA dual therapy was not inferior to VAC-triple therapy, and the low-dose vonoprazan group showed superior efficacy compared to the high-dose group. One possible explanation is that both vonoprazan and clarithromycin are metabolized by the same hepatic enzyme (cytochrome P450 3A4), so triple therapy can increase the maximum plasma concentration and the area under the plasma concentration–time curve of vonoprazan [29]. Additionally, *H. pylori* grows at a narrow external pH range between 6 and 7 and is sensitive to growth-dependent antibiotics, including amoxicillin. The bacterium grows poorer at pH 7.9 than at pH 7.2 [30]. Therefore, it results in a decrease in *H. pylori* sensitivity to amoxicillin in triple therapy [14]. Hence, low doses of vonoprazan (20 mg, qd) and a shorter treatment course (7 days) are recommended for the eradication of *H. pylori*. However, this finding still requires to be validated by further clinical trials.

There were certain limitations in this meta-analysis: (1) this study only included five English studies and one Chinese study, which might lead to language bias due to the lack of literatures in other languages; (2) positive results are more likely to be published than negative results, and the degree of publication bias is uncertain due to the limited number of studies; and (3) from a clinical perspective, as long as the effect of VA therapy is not inferior to that of triple or quadruple therapy and the rate of adverse reactions is similar, VA therapy can be considered to be clinically significant. However, it will lead to a combined effect size (RR) that is very close to the invalid line, thereby affecting the results of the meta-analysis. Recently, more than 10 ongoing randomized controlled trials on VA therapy are identified in the Cochrane Library; these trials will provide useful findings in the future.

VA dual therapy has shown a curative effect and acceptable safety in the treatment of *H. pylori* infection when compared with PPI-based triple therapy. Better compliance of patients can be achieved by reducing drug dosage and resistance. Can VA dual therapy be a new acceptable treatment for *H. pylori* infection? One RCT [8] showed that vonoprazan-based triple therapy was significantly superior to PPI-based therapy for patients with clarithromycin-resistant strains (eradication rates, 82.0% vs. 40.0%; 95% CI 3.63–12.86; *p* < 0.0001) if it could be used as a salvage therapy after failure of conventional PPI eradication therapy which was along with our results. Besides, the efficacy of VA dual therapy was comparable with VAC triple therapy, which was also consistent with the results of Ouyang et al. [24].

Therefore, we recommend VA dual therapy as an alternative method for *H. pylori* eradication which was better than traditional PPI-based triple therapy. Due to the limited inclusion, this result required more RCTs to clarify the eradication effect in the future. Meanwhile, the relationship between dose and duration of VA dual therapy and eradication deserved further exploration.

### Supplementary Information

**Additional file 1.** The search strategy of each database.

## Data Availability

The original contributions presented in the study are included in the article, further inquiries can be directed to the corresponding author.
