# Supplementary material for: The efficacy and safety of vonoprazan–amoxicillin dual therapy in eradicating Helicobacter pylori: a systematic review and meta-analysis
Source: Eur J Med Res. 2023 Aug 7;28:272. doi: 10.1186/s40001-023-01249-6 (PMC10405488; doi:10.1186/s40001-023-01249-6)
Supplement: Supplementary file 1 — Additional file 1. The search strategy of each database. [file 40001_2023_1249_MOESM1_ESM.docx]

**Pubmed,n=33,2022.7.7**

#1"vonoprazan"[Title/Abstract] OR "tak-438"[Title/Abstract] OR "VPZ"[Title/Abstract] OR "potassium competitive acid blocker"[Title/Abstract]

#2"helicobacter pylori"[Title/Abstract] OR "h pylori"[Title/Abstract]

#3"dual" [All Fields]

#4=#1 and #2 and #3

**Cochrane Library ,n=45,2022.7.7**

#1(vonoprazan or tak-438 or VPZ or potassium competitive acid blocker):ab

#2 (helicobacter pylori or h pylori):ab

#3(dual)

#4=#1 and #2 and #3

**Embase, n=67,2022.7.7**

#1 'vonoprazan'

#3 vpz

#4 'potassium competitive acid blocker'

#5 'helicobacter pylori'

#6 'h pylori'

#7 dual

#8 #1 OR #2 OR #3 OR #4

#9 #5 OR #6

#10 #7 AND #8 AND #9

**CNKI(n=8),2022.7.7**

[( Abstract = vonoprazan) AND (Abstract = helicobacter pylori)](https://kns.cnki.net/kns8/AdvSearch?id=51&dbcode=CFLS&searchtype=gradeSearch&ishistory=1)

**Wanfang Database(n=7),2022.7.7**

[( Abstract = vonoprazan) AND (Abstract = helicobacter pylori)](https://kns.cnki.net/kns8/AdvSearch?id=51&dbcode=CFLS&searchtype=gradeSearch&ishistory=1)
